# Acute-on-Chronic Limb Ischemia Mimicking Cellulitis: A Diagnostic Pitfall

**DOI:** 10.7759/cureus.117131

**Published:** 2026-09-29

**Authors:** Saima Shahryar, Ahmer A Longi, Misbah Fazlani, Shaffuq Naz, Mekki Medani

**Affiliations:** 1 General Surgery, Mediclinic Welcare Hospital, Dubai, ARE; 2 Internal Medicine, Mediclinic Welcare Hospital, Dubai, ARE; 3 Vascular Surgery, Mediclinic Welcare Hospital, Dubai, ARE

**Keywords:** acute-on-chronic limb ischemia, arterial thrombosis, careful vascular examination, lower limb cellulitis, negative d-dimer, peripheral arterial disease (pad), preserved sensory and motor functions, timely vascular imaging

## Abstract

Acute-on-chronic limb ischemia may present with clinical features that overlap with inflammatory conditions, potentially delaying recognition of arterial compromise. We report a case of an elderly woman with long-standing polycythemia treated with hydroxyurea who presented with progressive right foot pain, marked swelling, erythema, tenderness, and bluish discoloration of the hallux. She was discharged from the emergency department but returned the next night with worsening pain. Soft-tissue ultrasonography demonstrated diffuse subcutaneous edema without a localized collection, supporting an initial provisional diagnosis of cellulitis. Despite preserved motor function and sensation, progressive ischemic discoloration, prolonged capillary refill, and abnormal distal pulse findings prompted further vascular assessment. Computed tomography angiography (CTA) demonstrated a 6.2-cm right popliteal arterial thrombus with impaired distal perfusion. Her D-dimer was within the laboratory reference range at 0.40 µg/mL FEU, while the hematocrit was markedly elevated at 55.3%. Intravenous unfractionated heparin was initiated, resulting in improvement in pain and swelling and clinical stabilization, while definitive revascularization options were considered. The patient subsequently elected to pursue definitive treatment in her home country, precluding assessment of long-term limb-salvage outcomes. This case emphasizes the importance of careful and repeated vascular assessment in a painful, discolored limb, particularly before neuromotor deficits develop. Laboratory findings, including D-dimer, should be interpreted within their validated clinical context and should not supersede clinical evidence of impaired arterial perfusion.

## Introduction

Acute limb ischemia (ALI) is a sudden reduction in arterial blood flow to a limb that may threaten tissue viability and requires prompt recognition and management [1,2]. It commonly presents with pain, pallor or discoloration, reduced or absent pulses, sensory disturbance, and motor weakness as ischemia progresses [1,2]. Acute-on-chronic limb ischemia refers to an acute deterioration in perfusion occurring in a limb with pre-existing peripheral arterial disease. In contrast, chronic limb-threatening ischemia (CLTI) is a chronic clinical syndrome requiring appropriate clinical and objective evidence of peripheral arterial disease [3]. In the present case, objective hemodynamic measurements were not available to establish CLTI definitively; therefore, the presentation is described as acute-on-chronic limb ischemia.

Popliteal artery thrombosis refers to thrombotic obstruction of the popliteal artery and may substantially reduce blood flow to the lower leg and foot. Acute arterial thrombosis may occur in the setting of underlying peripheral arterial disease and can result in ALI [1,2,4]. Recognition may be challenging when ischemia presents with swelling, erythema, warmth, and tenderness because these findings can overlap clinically with cellulitis. Careful vascular examination is therefore essential in a painful or discolored limb. Assessment of distal pulses, capillary refill, skin color and temperature, sensation, and motor function can identify arterial compromise and help determine the urgency of management [1,2].

D-dimer is a fibrin degradation product released when cross-linked fibrin within a blood clot is broken down. It is well established as part of diagnostic strategies for excluding venous thromboembolism in appropriately selected patients [5-7]. In patients with peripheral arterial disease or peripheral arterial occlusive disease, elevated D-dimer concentrations have been associated with subsequent cardiovascular and arterial thrombotic events [8,9]. However, current consensus guidance emphasizes that such associations do not establish a sufficiently defined role for routine D-dimer testing in individual patients with arterial disease [10]. Accordingly, D-dimer should be interpreted within its validated clinical context and should not supersede concerning vascular examination findings when arterial compromise is suspected.

This case report describes an elderly woman with long-standing polycythemia who developed acute-on-chronic limb ischemia due to right popliteal arterial thrombosis after an initial presentation that clinically and ultrasonographically suggested cellulitis. The case highlights the diagnostic overlap between inflammatory and ischemic presentations and emphasizes the importance of repeated vascular assessment and timely arterial imaging when clinical findings raise concern for arterial compromise.

## Case presentation

An 82-year-old woman presented with a several-week history of right foot pain and swelling, predominantly involving the great toe, with significant worsening of the pain over the preceding two days. She had a history of recent travel. Four months before the current presentation, she had sustained blunt trauma to the right foot with a suspected fracture. She was treated conservatively without immobilization, continued to weight-bear, and the pain was tolerable at that time. Over the month preceding presentation, she developed increasing pain and marked swelling of the great toe.

At her first emergency department presentation at night (day 1), the right foot demonstrated marked swelling and erythema over the dorsum, with severe tenderness of the great toe and bluish discoloration of the toes. There was no calf swelling or tenderness. The foot was not cold, and motor function and sensation were preserved. The dorsalis pedis pulse was not palpable clinically in the presence of marked swelling; however, handheld Doppler examination demonstrated a positive dorsalis pedis arterial signal.

The patient returned to the emergency department the following night (day 2) with worsening pain. She was afebrile. In view of the prominent swelling, erythema of the right foot, and marked great-toe tenderness, cellulitis or a localized abscess involving the great-toe region was suspected. Soft-tissue ultrasonography of the right foot was therefore performed (Figure 1). This demonstrated diffuse subcutaneous thickening and hypoechogenicity consistent with edema and cellulitic changes, without a large localized superficial collection. She was admitted with a provisional diagnosis of right foot cellulitis and commenced on intravenous clindamycin 600 mg every eight hours.

**Figure 1:**
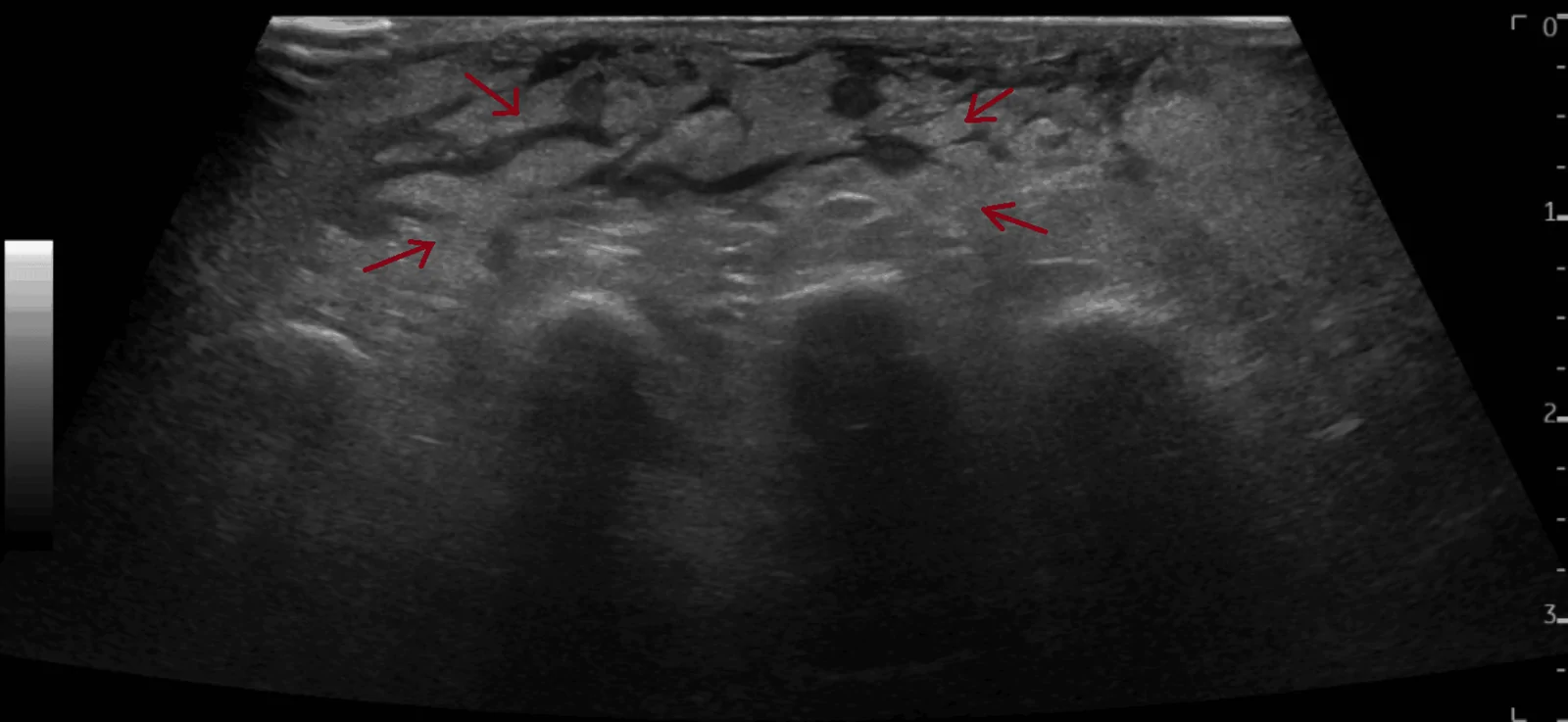
Ultrasound of the soft tissues of the dorsum of the right foot. Ultrasound demonstrating diffuse subcutaneous thickening and hypoechoic fluid tracking within the subcutaneous tissues, consistent with edema and cellulitic changes. No large localized superficial collection was identified. Red arrows indicate subcutaneous thickening and hypoechoic fluid tracking within the subcutaneous tissues.

Past medical history

Her medical history included right foot trauma approximately four months earlier, resulting in a fracture that had been managed conservatively abroad; long-standing polycythemia treated with hydroxyurea for more than 10 years; hypertension; and a previous deep vein thrombosis approximately 10 years earlier. She was an ex-smoker with an estimated 10 pack-year smoking history. There was no known history of diabetes mellitus, atrial fibrillation, ischemic heart disease, cerebrovascular disease, or previous peripheral arterial intervention.

Clinical examination

At the initial presentation, the right foot was markedly swollen and erythematous, with tenderness and bluish discoloration of the hallux. Over the subsequent clinical course, the hallux discoloration progressed to a blackish, dry necrotic appearance. The clinical photograph obtained on day 3 demonstrates this later appearance rather than the initial cellulitis-like presentation (Figure 2). Ischemic change involving the fifth toe was documented clinically but was not clearly appreciable in the available day 3 photograph.

**Figure 2:**
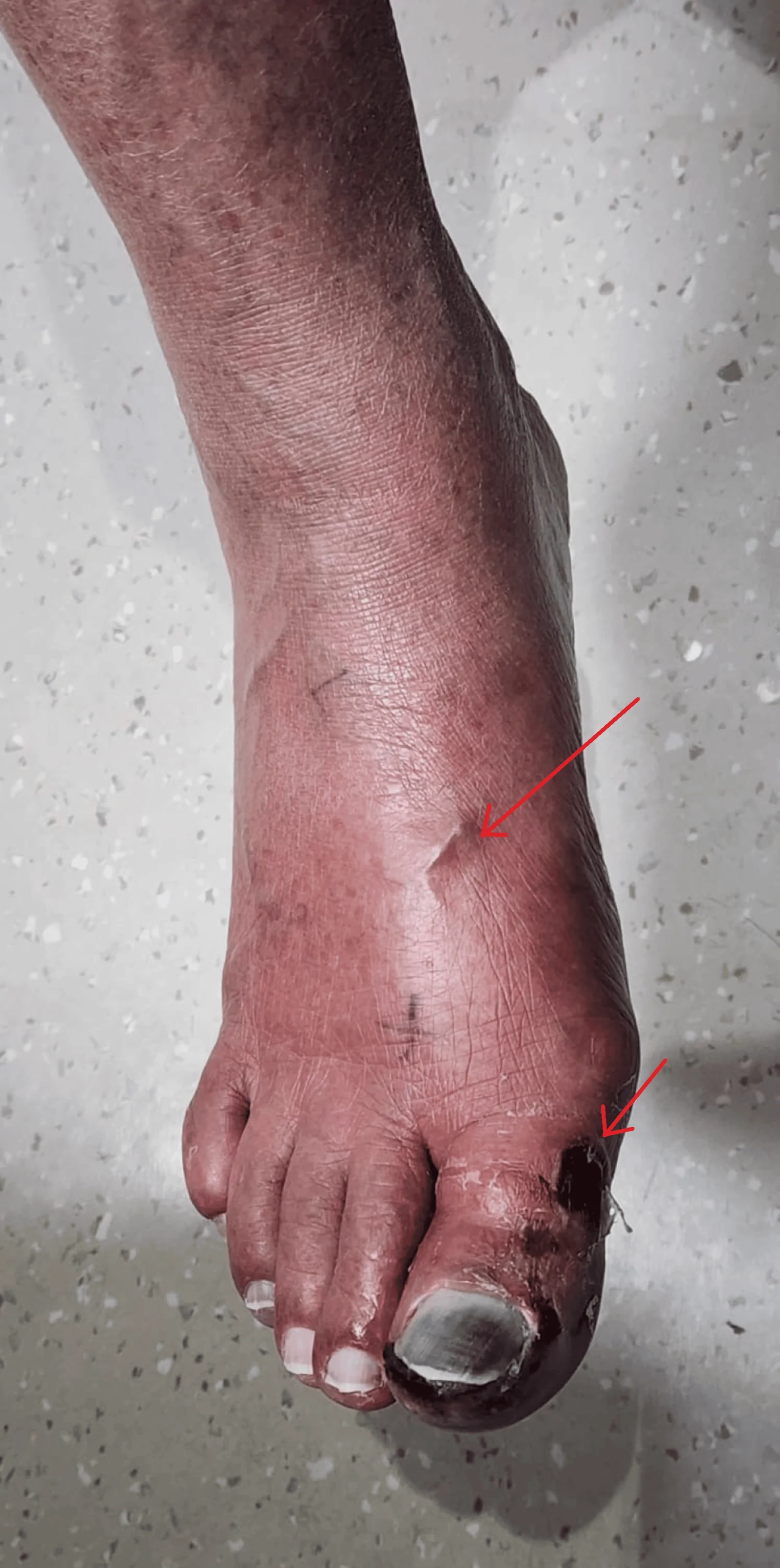
Clinical appearance of the right foot. Clinical photograph demonstrating an erythematous forefoot with swelling and bluish-black discoloration of the hallux. The upper red arrow indicates forefoot swelling and erythema, while the lower red arrow indicates discoloration involving the hallux with an associated blackish necrotic patch.

With progression of symptoms, examination demonstrated markedly prolonged capillary refill and abnormal distal pulse findings in the right lower limb, while femoral pulses remained palpable bilaterally. Importantly, motor function and sensation remained preserved despite the evolving ischemic findings. The combination of worsening pain, progressive discoloration, impaired capillary perfusion, and abnormal distal pulse findings prompted reconsideration of the initial inflammatory diagnosis and further vascular assessment.

On admission, the patient was hemodynamically stable, with vital signs as summarized in Table 1.

**Table 1:** Vital signs on admission. SpO_2_: peripheral capillary oxygen saturation

Parameter	Value
Blood pressure	125/69 mmHg
Pulse rate	87 beats/min
Respiratory rate	18 breaths/min
SpO₂	98% on room air
Temperature	36.8°C

Serial laboratory investigations

The initial coagulation profile showed an international normalized ratio (INR) of 1.05 and activated partial thromboplastin time (APTT) of 35.6 seconds. Notably, the D-dimer was within the laboratory reference range at 0.40 µg/mL FEU (reference 0.27-0.80 µg/mL FEU). These findings are summarized in Table 2. The day 4 APTT of 62.5 seconds was measured after initiation of intravenous unfractionated heparin and should therefore be interpreted in the context of anticoagulant therapy.

**Table 2:** Consolidated laboratory investigations. Other investigations included a hemoglobin A1c level of 6.2% and a B-type natriuretic peptide level of 95 pg/mL on day 2. Liver function tests were performed once on day 4: total bilirubin was 27.8 µmol/L, direct bilirubin was 11.7 µmol/L, alkaline phosphatase was 144 U/L, alanine aminotransferase was 24.3 U/L, aspartate aminotransferase was 25.4 U/L, albumin was 30.3 g/L, and total protein was 64 g/L. WBC: white blood cell count; eGFR: estimated glomerular filtration rate; CRP: C-reactive protein; APTT: activated partial thromboplastin time; PT: prothrombin time; INR: international normalized ratio

Investigation	Day 1	Day 2	Day 3	Day 4	Day 5	Units	Reference Range
WBC	10.5	-	8.2	-	-	×10⁹/L	4.0-11.0
Platelet count	292	-	292	-	-	×10⁹/L	150-450
Hemoglobin	17.6	-	16.0	-	-	g/dL	11.5-16.0
Hematocrit	55.3	-	51	-	-	%	31-42
CRP	123	-	103	-	-	mg/L	0-5
Creatinine	94	-	-	69.9	-	µmol/L	53-106
eGFR	52	-	-	75	-	mL/min/1.73 m²	>60
D-dimer	-	0.40	-	-	-	µg/mL FEU	0.27-0.80
APTT	35.6	-	38.4	62.5	65.6	seconds	28.6-40.0
PT	14.7	-	-	-	-	seconds	11.7-15.3
INR	1.05	-	-	-	-	-	0.80-1.20

Differential diagnosis

The initial combination of swelling, erythema, tenderness, elevated inflammatory markers, and ultrasonographic edema supported a provisional diagnosis of cellulitis. However, the progressive toe discoloration, markedly prolonged capillary refill, worsening pain, and abnormal distal pulse findings raised increasing concern for arterial compromise. Acute-on-chronic limb ischemia became the principal consideration. Blue toe syndrome and vasculitis were also considered in the differential diagnosis; however, a dedicated workup for these conditions was not documented. Computed tomography angiography (CTA) subsequently provided a direct anatomical explanation for the ischemic presentation by demonstrating right popliteal arterial thrombosis.

Management

During admission, the clinical appearance evolved despite treatment. By day 3, the hallux demonstrated progression from the bluish discoloration noted at presentation to blackish, dry necrotic change. Ischemic change of the fifth toe was also documented clinically (Figure 2). Capillary refill time was approximately five seconds on the right compared with less than three seconds on the left. On subsequent vascular examination, the dorsalis pedis and popliteal pulses were not palpable on the right side. Motor function and sensation remained preserved.

Progression of the ischemic findings, including worsening pain and discoloration with development of necrotic change and a hemorrhagic bulla, prompted urgent CTA and vascular surgical consultation. CTA demonstrated a 6.2-cm long thrombotic occlusion of the right popliteal artery, with distal contrast filling of the anterior tibial, posterior tibial, and fibular arteries. Reduced distal opacification was noted in portions of the right infra-popliteal vessels, while the right dorsalis pedis and plantar arteries remained contrast-filled. Associated soft-tissue edema was present over the dorsal and plantar aspects of the right foot (Figure 3). These findings were consistent with acute-on-chronic limb ischemia secondary to popliteal artery thrombosis.

**Figure 3:**
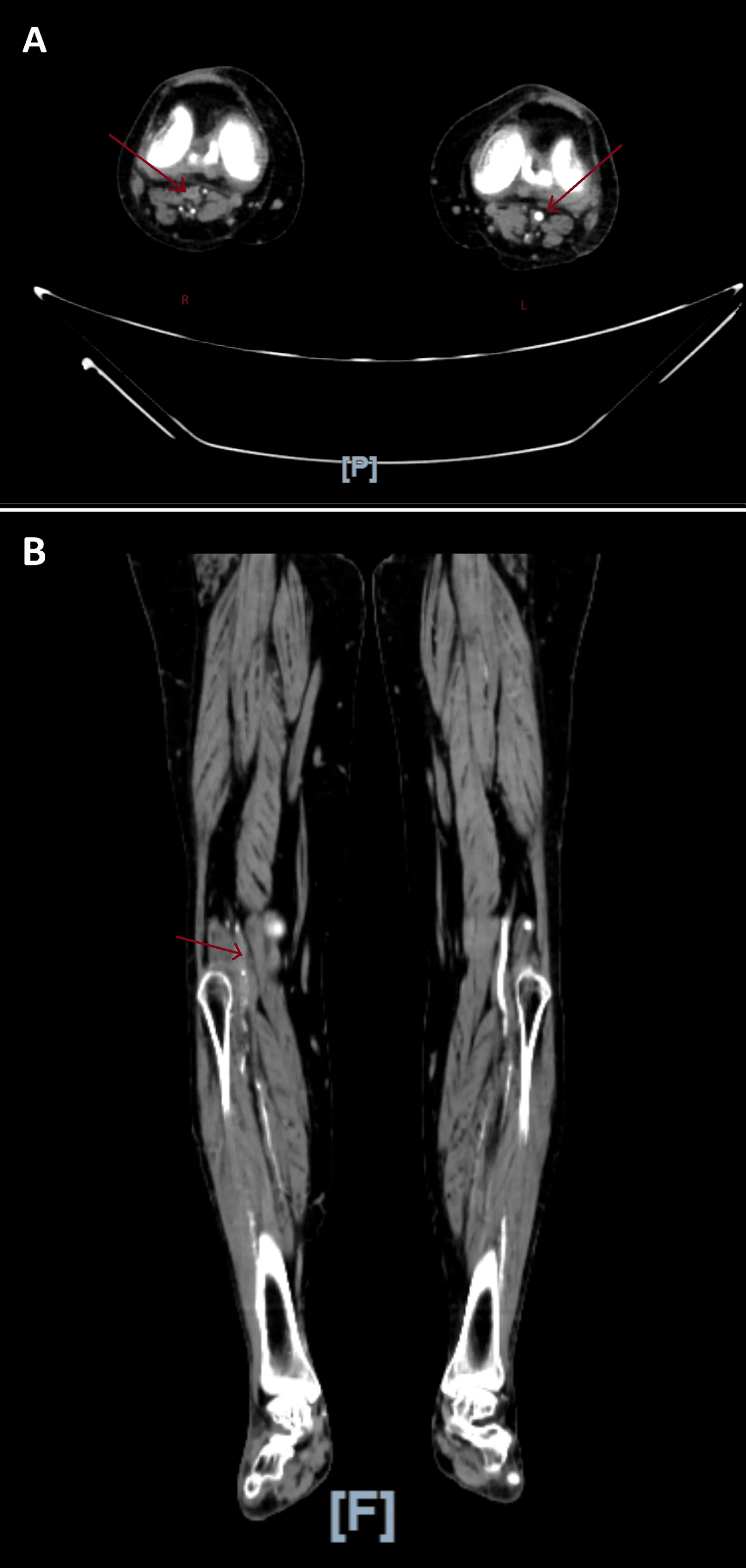
Computed tomography angiography of the lower limbs. (A) Axial view. The left arrow indicates the visualized left popliteal artery, while the right arrow demonstrates the thrombosed/non-opacified right popliteal artery. (B) Coronal view demonstrating thrombosis of the right popliteal artery measuring approximately 6.2 cm, with associated soft-tissue edema involving the dorsum and plantar aspect of the right foot. The red arrow indicates the site of right popliteal artery occlusion.

The patient was subsequently transferred to the ICU and commenced on intravenous unfractionated heparin infusion with a target APTT of 60-80 seconds. Analgesia was provided with a fentanyl infusion. Vascular surgery and hematology teams were involved in multidisciplinary management. 

Detailed discussions were held by the vascular surgeon with the patient and her family regarding the diagnosis of limb-threatening ischemia, prognosis, and available treatment options. Potential interventions including open thrombectomy with or without bypass and endovascular revascularization were discussed, together with the associated risks and benefits.

Hematology review recommended continuation of heparin therapy and hydroxyurea. Additional investigations for JAK2 mutation and erythropoietin levels were deferred as they were not expected to alter immediate management. Therapeutic phlebotomy was not recommended because of concerns regarding compromised limb perfusion.

Final diagnosis

Following clinical reassessment and vascular imaging, the final diagnosis was acute-on-chronic right lower-limb ischemia secondary to a 6.2-cm right popliteal artery thrombus.

Outcome and follow-up

This case report is limited to the index admission only. The patient remained clinically stable after anticoagulation, with improvement in pain and swelling, preserved motor and sensory function, and detectable distal Doppler signals. No follow-up vascular imaging was performed at our institution as the patient was self-pay and proceeded to continue definitive treatment in her home country; the subsequent revascularization strategy and long-term limb-salvage and functional outcomes are unknown.

She was discharged against medical advice (DAMA) on a therapeutic dose of low-molecular-weight heparin (Clexane 70 mg twice daily subcutaneous injection), oral clindamycin 300 mg thrice daily, tramadol 100 mg tablet four times daily along with co-codamol thrice daily for breakthrough pain, besides instructions to continue her chronic medications and other supportive medications for acidity and nausea.

The patient and family were instructed to proceed directly to a hospital upon arrival in their country for definitive management.

## Discussion

This case illustrates the diagnostic challenge of acute-on-chronic limb ischemia presenting with inflammatory features that initially suggested cellulitis. Marked swelling, erythema, tenderness, elevated inflammatory markers, and ultrasonographic evidence of subcutaneous edema contributed to the initial provisional diagnosis. However, these findings were nonspecific and could also occur in association with ischemic tissue injury. The subsequent clinical course, including persistent and worsening pain, progressive toe discoloration, prolonged capillary refill, and abnormal peripheral pulse findings, increased concern for impaired arterial perfusion and prompted arterial imaging.

D-dimer is a degradation product of cross-linked fibrin and is well established in diagnostic algorithms for venous thromboembolism in appropriately selected patients [4-6,10]. Its diagnostic role in peripheral arterial thrombosis, however, is not established. The D-dimer level in this patient was 0.40 µg/mL FEU, within the laboratory reference range of 0.27-0.80 µg/mL FEU. This finding is best regarded as a secondary laboratory observation rather than a means of excluding arterial thrombosis. Although the duration of symptoms raises the possibility of an evolving or organized thrombotic process, neither the age nor histological composition of the thrombus was directly assessed. Therefore, the normal D-dimer cannot be attributed definitively to thrombus organization, diminished fibrinolysis, or thrombus composition.

An additional prothrombotic consideration was the patient's long-standing polycythemia, treated with hydroxyurea for more than 10 years, with a hematocrit of 55.3% at presentation. Polycythemia is associated with increased thrombotic risk, and evidence in polycythemia vera supports maintaining hematocrit below 45% to reduce cardiovascular death and major thrombosis [11,12]. Hydroxyurea is used as cytoreductive therapy to reduce thrombotic risk rather than being considered a cause of thrombosis [12]. The elevated hematocrit was therefore a clinically relevant prothrombotic factor that may have contributed to the arterial event. Nevertheless, the mechanism of the popliteal thrombosis cannot be established definitively from this case, and an embolic mechanism cannot be excluded.

Clinical vascular examination remains central when limb ischemia is suspected. At the initial emergency department assessment, the dorsalis pedis pulse was not palpable in the setting of marked foot swelling; however, a positive dorsalis pedis arterial signal was detected with handheld Doppler. On subsequent examination, capillary refill on the affected right side was approximately five seconds compared with less than three seconds on the left. These findings demonstrate why pulse palpation should be interpreted alongside Doppler assessment, capillary refill, skin appearance, temperature, sensation, and motor function. Inflammatory features and laboratory results should not supersede abnormal findings of peripheral perfusion.

Preserved motor and sensory function should not provide false reassurance when other findings suggest impaired arterial perfusion. Neuromotor deficits generally indicate more advanced limb threat; therefore, clinical vigilance and serial vascular assessment are particularly important while the limb remains viable and before such deficits develop. In this patient, motor function and sensation remained preserved despite progressive ischemic discoloration and arterial occlusion, emphasizing the need to respond to evolving perfusion abnormalities before neurological deterioration occurs.

CTA demonstrated a 6.2-cm right popliteal arterial thrombus/occlusion with impaired distal perfusion, providing an anatomical explanation for the clinical findings. The presentation is most appropriately described as acute-on-chronic limb ischemia because an acute thrombotic event occurred in the setting of underlying chronic peripheral arterial disease. Quantitative hemodynamic measurements such as ankle-brachial index and toe pressures were not available; therefore, CLTI was not retained as the definitive diagnostic terminology.

The initial toe discoloration also raised consideration of other ischemic causes, including distal embolic or blue toe phenomena and vasculitis. However, CTA demonstrated a substantial right popliteal arterial occlusion with impaired distal perfusion, providing a direct anatomical explanation for the limb ischemia. No systemic clinical features specifically suggesting vasculitis were documented. Because a complete etiologic evaluation for vasculitis or an embolic source was not completed during the index admission, these mechanisms cannot be considered definitively excluded.

Intravenous unfractionated heparin was initiated to reduce further thrombus propagation while limb perfusion was monitored. Pain and swelling improved during the index admission, distal Doppler signals remained detectable, and no motor or sensory deficit developed. This represented short-term clinical stabilization rather than definitive treatment of the arterial occlusion. Definitive management and long-term outcome could not be assessed because the patient elected to continue treatment in her home country.

Limitations

This case has several limitations. Post-discharge follow-up was unavailable because the patient elected to continue definitive treatment in her home country; therefore, subsequent revascularization details, long-term limb-salvage outcome, and functional recovery are unknown. Quantitative hemodynamic measurements, including ankle-brachial index and toe pressures, were not obtained. In addition, JAK2 mutation testing was not performed during the index admission because it was not expected to alter immediate management, limiting confirmation of the precise hematologic subtype of the documented polycythemia.

## Conclusions

This case demonstrates that acute-on-chronic limb ischemia can initially resemble cellulitis, particularly when swelling, erythema, tenderness, and inflammatory changes predominate. Clinical deterioration despite treatment for an inflammatory process should prompt reconsideration of the diagnosis and assessment for an underlying vascular cause. Early recognition of evolving ischemia through repeated vascular examination and timely vascular imaging is important to establish the diagnosis and guide appropriate management.
